# METHODOLOGICAL AND ETHICAL REFLECTIONS FROM RESEARCH ON NEIGHBOURHOOD-BUILT ENVIRONMENT AND DEMENTIA

**DOI:** 10.1093/geroni/igac059.883

**Published:** 2022-12-20

**Authors:** Kishore Seetharaman, Habib Chaudhury, Lillian Hung, Shannon Freeman, Mark Groulx, Cari Randa

**Affiliations:** Simon Fraser University, Vancouver, British Columbia, Canada; Simon Fraser University, Vancouver, British Columbia, Canada; University of British Columbia, Vancouver, British Columbia, Canada; University of Northern British Columbia, Prince George, British Columbia, Canada; University of Northern British Columbia, Prince George, British Columbia, Canada; Simon Fraser University, Vancouver, British Columbia, Canada

## Abstract

The neighbourhood is widely regarded as a setting that affords emotional and practical support and opportunities to maintain community-based activities and social participation for people living with dementia (PLWD). Creating a supportive neighbourhood built environment that facilitates outdoor mobility, wayfinding, and access to community destinations is key to making our communities dementia-inclusive. Research on the built environment and dementia-inclusive planning is relatively sparse in the broader research domain of neighbourhoods and dementia. Further, how PLWD perceive, interpret, and interact with the neighbourhood built environment is not adequately understood. Although it is acknowledged that PLWD should be more meaningfully included and engaged in research in this area, there is a lack of guidance on methodological and ethical considerations necessary to explore people-place relations in the neighbourhood built environment through the lens of the lived experience of PLWD. To address this gap, our paper draws from the Dementia-inclusive Streets and Community Access, Participation, and Engagement (DemSCAPE) study to highlight reflections on conducting walk-along interviews, embodied videography, photo documentation, semi-structured sit-down interviews, and visual elicitation to explore the influence of the neighbourhood built environment on the outdoor walking experience of PLWD. We discuss 1) methodological strengths, including the triangulated strategy of capturing experiential data in-situ in real time and in retrospect, and flexibly working around memory and communication-related challenges experienced by PLWD, 2) ethical challenges and measures for mitigation, 3) logistical difficulties in undertaking complex fieldwork with PLWD, limitations of the research methods, and potential alternative methods to explore in future research.

